# Subwavelength broadband sound absorber based on a composite metasurface

**DOI:** 10.1038/s41598-020-70714-7

**Published:** 2020-08-14

**Authors:** Houyou Long, Chen Liu, Chen Shao, Ying Cheng, Kai Chen, Xiaojun Qiu, Xiaojun Liu

**Affiliations:** 1 Key Laboratory of Modern Acoustics, Institute of Acoustics, Nanjing University, Nanjing, 210093 China; 2 Centre for Audio, Acoustics and Vibration, Faculty of Engineering and IT, University of Technology Sydney, Sydney, NSW 2007 Australia

**Keywords:** Applied physics, Acoustics

## Abstract

Suppressing broadband low-frequency sound has great scientific and engineering significance. However, normal porous acoustic materials backed by a rigid wall cannot really play its deserved role on low-frequency sound absorption. Here, we demonstrate that an ultrathin sponge coating can achieve high-efficiency absorptions if backed by a metasurface with moderate surface impedance. Such a metasurface is constructed in a wide frequency range by integrating three types of coiled space resonators. By coupling an ultrathin sponge coating with the designed metasurface, a deep-subwavelength broadband absorber with high absorptivity (${>}80\%$) exceeding one octave from  185 Hz to  385 Hz (with wavelength $\lambda$ from 17.7 to 8.5 times of thickness of the absorber) has been demonstrated theoretically and experimentally. The construction mechanism is analyzed via coupled mode theory. The study provides a practical way in constructing broadband low-frequency sound absorber.

## Introduction

Traditional absorbers for airborne sound, i.e., porous material layer facing free space on one side and backed by a rigid wall on the other side, rely on the viscous dissipation and heat conduction for sound energy consumption. Such sandwich architecture behaves as an Fabry-Perot-like cavity and consequently, the sound waves with wavelength smaller than 4 times of material thickness can be efficiently consumed, bringing about the inherent limitations of heavy and bulky treatments to dissipate long-wavelength sound^1^. To improve the low-frequency sound absorption, micro-perforated panels (MPPs)^2^ characterized as subwavelength-dimensional absorber have been devised. However, the total thicknesses of MPPs are still not subwavelength enough due to the necessary backing cavity and show limitation of the narrow working frequency band, which consequently act as sound facings generally^3^ . To further reduce the thickness of the sound absorber, great efforts have been devoted to pursue new candidates to construct impedance-matched surface based on metamaterials resonators^4–21^ which are essential to achieve perfect absorption (PA). Due to the highly concentrated sound energy at resonance, very small dissipation coefficients, i.e., viscosity from surface tensor in membrane and fractional viscosity between air and framework in fluid-solid system, can achieve high-efficiency or even PA. However, the highly concentrated sound energy in resonant system also results in a little sound energy radiated^22^, which induces the sharp absorptive peak associated with a small radiation or leakage factor. Hence, most of these absorbers generally work at a single or multiple discrete narrow bands.

To extend the working frequency range, multiple resonant unit cells are generally required to be integrated, i.e., broadband absorber based on labyrinthine acoustic metamaterials^19^. Unfortunately, achieving broader bandwidth and thinner device are contradict. Regarding coupled-mode theory (CMT), to achieve larger radiation loss (indicating broader bandwidth), the total thickness should be increased^22^. Moreover, due to the sharp absorptive peak of individual unit cell, the absorptance of the combined system is bound to emerge low-efficiency absorption valleys at some extent. Recently, based on causality principle^23–25^, optimal sound absorber^20^ showing highly efficient absorption at full frequency above the low-frequency cutoff frequency (with a thickness of $\lambda /10$) have been proposed. The ingenuity of the strategy is letting the system simultaneously dissipates the propagated and evanescent sound wave components to approach near-unity absorption. To further lower the working frequency, the absorptive metasurface showing broadband near-unity absorption at deep sub-wavelength dimension (with a wavelength $\lambda$ being from 12.6 to 9.0 times of the thickness at absorptance $>95\%$) has been devised^22^. However, the bandwidth of absorption is still limited, i.e., less than one octave.

In this work, we further draw out the physical mechanism for broadband sound absorption from the view of impedance. A sub-wavelength composite metasurface is constructed by assembling an ultra-thin sponge and a backing metasurface with moderate surface impedance, which can achieve $>80\%$ absorption at frequencies exceeding one octave (from $\sim$185 Hz to $\sim$385 Hz, with wavelength being from $\sim$17.7 to $\sim$8.5 times of the thickness). In order to significantly enhance the coupling with the ultrathin sponge coating, the backing metasurface is integrated by multiple coiled space resonators (CSRs), which show a perfectly-matched impedance at 12 discrete frequencies and near-perfectly-matched impedances in the entire intervening frequency ranges. The absorption efficiency is largely enhanced in comparison with the case of rigid wall backing, whereas the absorptive bandwidth is extended when compared with the case of soft boundary backing^22^. In this work, the theoretical complex frequency planes calculated with the admittance-sum method and transfer-matrix method have been further employed to analyze the absorptive performances.

## Results

### Absorption of sponge coating with backing plate

**Figure 1:**
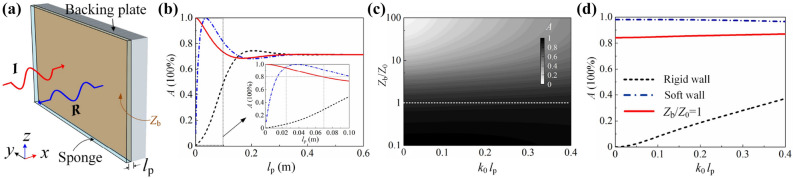
(**a**) Schematics of a sponge coating backed by a plate with surface impedance ${Z}_{\mathrm{b}}$ . (**b**) Absorptance of specific cases with ${Z}_{\mathrm{b}}/{Z}_{\mathrm{0}}\rightarrow \infty$ (black dashed line), ${Z}_{\mathrm{b}}/{Z}_{\mathrm{0}}\rightarrow 0$ (blue dash-dotted line) and ${Z}_{\mathrm{b}}/{Z}_{\mathrm{0}}=1$ (red solid line) at 200 Hz along with $l_{\mathrm{p}}$. (**c**) Absorptance distribution along with ${k}_{\mathrm{0}}{l}_{\mathrm{p}}$ at different normalized surface impedance of the backed plate ${Z}_{\mathrm{b}}/{Z}_{\mathrm{0}}$. (**d**) Absorptance of specific cases with sponge thickness at $l_{\mathrm{p}} =0.05 \, \hbox {m}$.

We start from an ideal model composed of an ultrathin coating layer of porous material (sponge for illustration) and a hypothetical backing plate with arbitrarily-tunable surface impedance ${Z}_{\mathrm{b}}$, as shown in Fig. 1a. Moreover, the backing plate prevents the sound energy from transmitting into the exit terminal. The thickness of the sponge coating is ${l}_{\mathrm{p}}$; the effective mass density $\rho _{\mathrm{p}}$ and compressibility modulus ${C}_{\mathrm{p}}$ can be given by the Johnson-Champoux-Allard (JCA) model (see “Methods” for details). The effective propagation constant and impedance are obtained as $k_{\mathrm{p}}=\omega \sqrt{(\rho _{\mathrm{p}} C_{\mathrm{p}})}$ and $Z_{\mathrm{p}}=\sqrt{(\rho _{\mathrm{p}}/C_{\mathrm{p}} )}$, respectively. The surface impedance of the system follows

1 $$\begin{aligned} Z_{\mathrm{S}}=Z_{\mathrm{p}} \frac{Z_{\mathrm{b}}+\mathrm{j}Z_{\mathrm{p}}\mathrm{tan}(k_{\mathrm{p}} l_{\mathrm{p}})}{Z_{\mathrm{p}}+\mathrm{j}Z_{\mathrm{b}} \mathrm{tan}(k_{\mathrm{p}} l_{\mathrm{p}})}. \end{aligned}$$

Thus, the absorptance can be determined as $A = 1-{\mid {(Z_{\mathrm{S}}-Z_0)}/{(Z_{\mathrm{S}}+Z_0)}\mid }^2$, where $Z_0={\rho _0 c_0}$ is the acoustic impedance of air medium. From Eq. (1), it is predicted that $Z_{\mathrm{S}} \approx Z_{\mathrm{b}}$ due to $\mathrm{tan}(k_{\mathrm{p}} l_{\mathrm{p}}) \approx 0$ in case with extremely thin sponge and consequently, acoustic characteristics of the system are dominated by the backing plate; to the contrary, performances are up to the over-thick sponge since $Z_{\mathrm{S}} \approx Z_{\mathrm{p}}$ because of $\mathrm{tan}(k_{\mathrm{p}} l_{\mathrm{p}}) \approx -j$ as illustrated in Fig. 1b, where the absorptance at 200 Hz (randomly selected) along with $l_{\mathrm{p}}$ is presented. Thus, the extreme cases are not considered and without loss of generality, $l_{\mathrm{p}}=0.05$ m is selected in this work. Figure 1c presents the absorptance distribution along with ${k}_{\mathrm{0}}{l}_{\mathrm{p}}$ at varied impedances of the backing wall, where ${k}_{\mathrm{0}}$ denotes the propagation constant in air medium. Excellent absorption performance at low frequency (${l}_{\mathrm{p}} < {\lambda }_{\mathrm{0}} = 2\pi /{k}_{\mathrm{0}}$) can be observed, which is gradually destroyed with the increasing ${Z}_{\mathrm{b}}$; namely, the absorptance is better with softer backing boundary.

In conventional manner, sound absorbers are composed of sponge coatings backed by a rigid wall. In this case, the reflected sound waves are in phase with the incident sound waves, leading to the sound pressure node formed at the surface of the backing plate. Consequently, the sound energy density inside the sponge coating is extremely low when ${l}_{\mathrm{p}} < {\lambda }_{\mathrm{0}}$, and a little sound energy can be dissipated (black dashed line in Fig. 1d) even the sponge coating possesses large viscosity and heat conduction coefficients. To the contrary, for the extreme case of acoustic soft-boundary with ${Z}_{\mathrm{b}}=0$, the sound pressure anti-node is formed in the vicinity of backing plate, leading to the destructive interference between reflected and incident waves. Thus, the sound energy is concentrated (twice amplitude of incident sound pressure) and can be dissipated by an ultrathin sponge, as illustrated by the blue dash-dotted line in Fig. 1d. Note that tunable in-between absorptance of $0<{A}<1$ can be obtained in corresponding cases of $0<{Z}_{\mathrm{b}}<\infty$ bounded by aforementioned extremely soft and rigid wall. Specifically, in the case of impedance-matching boundary with ${Z}_{\mathrm{b}}={Z}_0$, the system demonstrates >80% absorptance (red solid line in Fig. 1d). Although the system backed by an air slab presents ${Z}_{\mathrm{b}}={Z}_0$, the sound energy is hardly absorbed since which are largely transmitted when ${k}_{\mathrm{p}}{l}_{\mathrm{p}}\ll 1$. Furthermore, we emphasize that similar results can be achieved by porous materials with different flow resistivity (see “Methods” for details).

### Decorating impedance based on CSR

**Figure 2:**
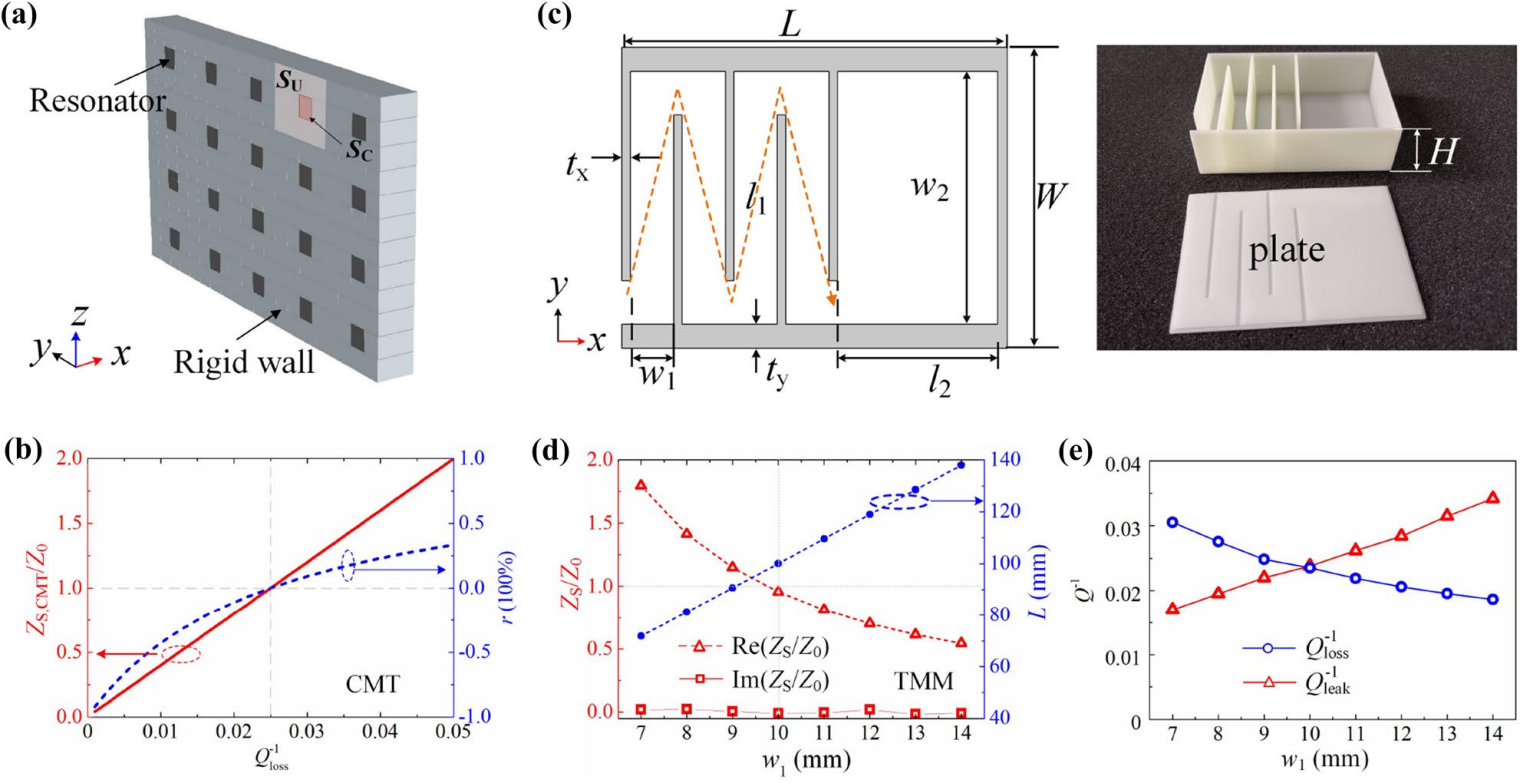
(**a**) Schematic diagram: the resonators are embedded into a rigid wall to achieve tunable surface impedance. (**b**) Surface impedance (red solid line) and reflection coefficient (blue dashed line) along with $Q_{\mathrm{loss}}^{-1}$ at $Q_{\mathrm{leak}}^{-1}=0.025$. (**c**) 2D schematics of CSR (left panel) and 3D photograph of a sample fabricated by 3D printing (right panel). (**d**) Surface impedance at resonant frequency (red symbol lines) of CSRs with different $w_1$. The blue symbol line indicates the variation of *L* to keep the resonant frequency at 236 Hz. (**e**) Retrieved loss and leakage factors.

Generally, it is difficult to find a natural materials “softer” than air, let alone for a broadband one. Instead, an acoustic resonant system is employed to decorate surface impedance as shown in Fig. 2a, in which the resonators are periodically embedded into a rigid wall in sparse pattern. According to CMT, the surface impedance of the system can be given as

2 $$\begin{aligned} Z_{\mathrm{S,CMT}}={\frac{-\mathrm{j}2(\omega /\omega _{\mathrm{r}}-1)+Q_{\mathrm{loss}}^{-1}}{Q_{\mathrm{leak}}^{-1}}}, \end{aligned}$$

where $\omega _{\mathrm{r}}=2 \pi f_{\mathrm{r}}$ is the resonant angular frequency of the resonator; $Q_{\mathrm{loss}}^{-1}$ is the loss factor responsible for energy dissipation due to viscosity and heat conduction; $Q_{\mathrm{leak}}^{-1}$ denotes the leakage factor responsible for energy leakage from the resonator to exterior space^26^. Specifically, the surface impedance can be simplified as $Z_{{\mathrm{S,CMT}}}=Q_{\mathrm{loss}}^{-1}/Q_{\mathrm{leak}}^{-1}$ at resonance ($\omega =\omega _{\mathrm{r}}$), which is a real number. Figure 2b demonstrates the surface impedance $Z_{{\mathrm{S,CMT}}}$ (red solid line) and reflection coefficient (blue dashed line) of the system with varied $Q_{\mathrm{loss}}^{-1}$ at fixed $Q_{\mathrm{leak}}^{-1}=0.025$. It is observed that the under-damped $(Q_{\mathrm{loss}}^{-1}<Q_{\mathrm{leak}}^{-1})$ system shows “soft” boundary effect while the over-damped one $(Q_{\mathrm{loss}}^{-1}>Q_{\mathrm{leak}}^{-1})$ do the opposite; the surface impedance will perfectly matched to that of air medium at $Q_{\mathrm{loss}}^{-1}=Q_{\mathrm{leak}}^{-1}$. Hence, a system satisfying under-damped, over-damped and critically-coupled condition can be treated as a acoustic soft, hard and impedance-matching boundary, respectively.

Here, CSR unit constructed by a thin zigzag channel (channel 1) and a wide straight channel (channel 2) is employed as the resonator. Figure 2c shows the cross-sectional view of a CSR sample with the corresponding geometric parameters annotated; $t_{{\mathrm{x}}}$ and $t_{{\mathrm{y}}}$ are the thicknesses of walls in *x* and *y* directions; $l_{{\mathrm{k}}}$ and $w_{{\mathrm{k}}}$ are length and width of the *k*-th ($k =1, 2$) channel; *W* and *H* are the width and height of the CSR. The inset shows the photograph of a CSR sample fabricated with 3D printing technology. The impedance can be derived from transfer-matrix method by characterizing the k-th channel with complex density $\rho _{\mathrm{e}}^{\mathrm{k}}$ and compressibility $C_{{\mathrm{e}}}^{\mathrm{k}}$ (see “Methods” for details).

**Figure 3:**
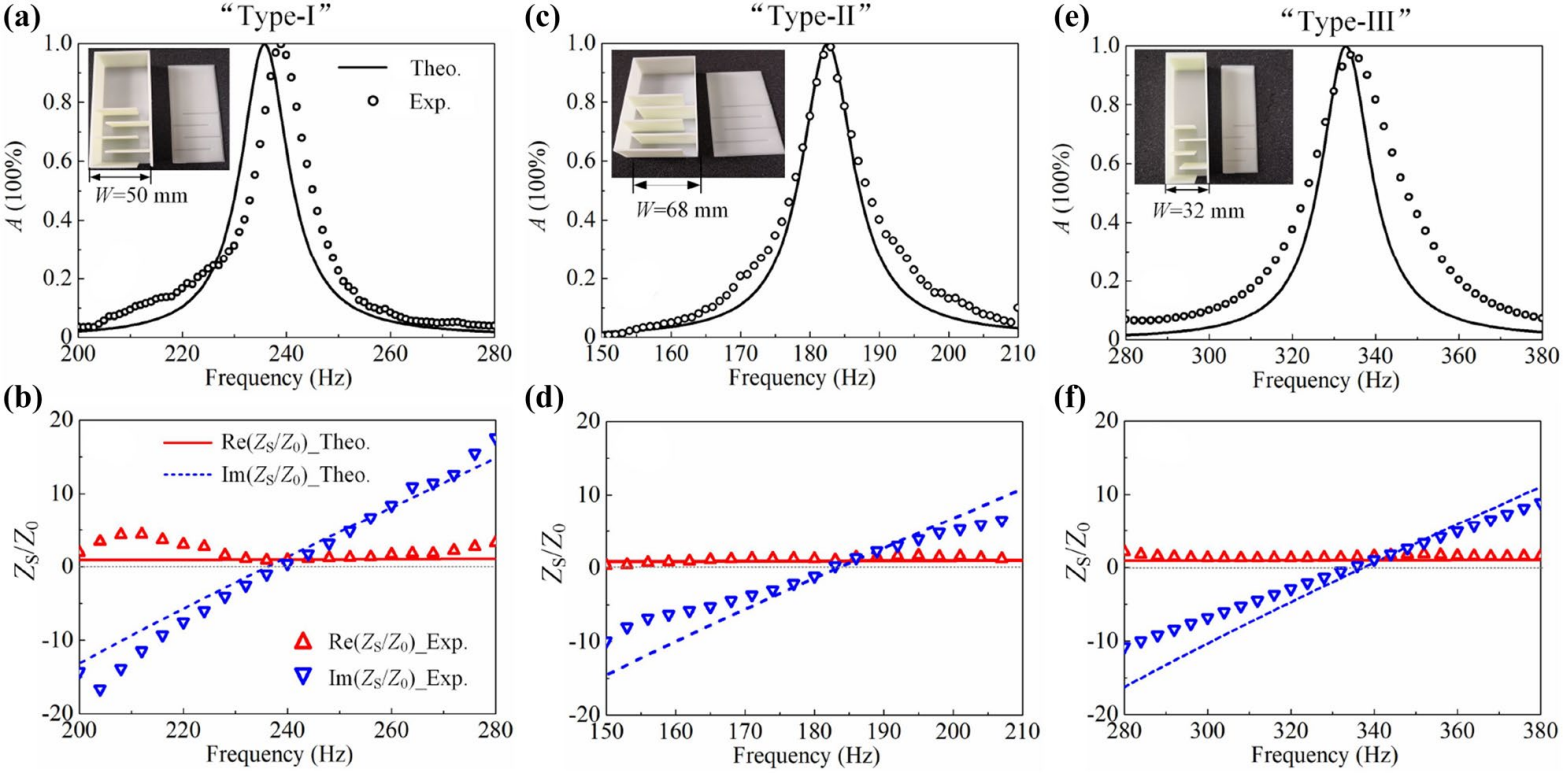
(**a**) Absorptance of metasurface absorber composed by type-I CSR at $w_1 =10 \, \hbox {mm}$, (**c**) by type-II CSR at $w_1 =11.4 \, \hbox {mm}$ and (**e**) by type-III CSR at $w_1 =8.6 \, \hbox {mm}$. (**b**), (**d**) and (**f**) are for the corresponding surface impedances.

We further construct a practical impedance metasurface based on CSRs with geometric parameters fixed at $W=50 \, \hbox {mm}$, $H =30 \, \hbox {mm}$, $t_{\mathrm{x}} =1.4 \, \hbox {mm}$, $t_{\mathrm{y}} =2.1 \, \hbox {mm}$, $N=4$ and $l_2=L-Nw_1-(N+2)t_{\mathrm{x}}$. Here, we have regulated the cross-section area of a unit cell and CSR as $S_{\mathrm{U}}=0.15\times 0.12 \, {\mathrm{m}}^{2}$ and $S_{\mathrm{C}}=0.05\times 0.03 \, {\mathrm{m}}^{2}$, respectively. Hence, the filling ratio of CSR is $r_{\mathrm{CSR}}=S_{\mathrm{C}}/S_{\mathrm{U}} =8.3\%$, indicating the CSR is sparsely distributed. Figure 2d shows the surface impedance variation (red lines with symbols) with different width of channel 1 ($w_1$) at the resonant frequency of 236 Hz. It can be observed that the imaginary parts of surface impedances approach zero at resonances while the real parts decrease along with $w_1$, indicating the effective boundary becomes softer. According to CMT, $Q_{\mathrm{loss}}^{-1}$ ($Q_{\mathrm{leak}}^{-1}$) decreases (increases) with the increase of $w_1$, which can be confirmed by the retrieved $Q_{\mathrm{loss}}^{-1}$ and $Q_{\mathrm{leak}}^{-1}$ shown in Fig. 2e. This is because the dissipation loss originates from channel 1, and increasing $w_1$ releases the viscous and heat-conducted dissipation. Simultaneously, increasing $w_1$ guarantees more sound energy leak from CSR to exterior space and induces the larger $Q_{\mathrm{leak}}^{-1}$. The impedance at $w_1=10 \, \hbox {mm}$ is perfectly matched to that of air and the system is in critically coupled state. However, to achieve a softer boundary at a fixed resonant frequency, it is necessarily to enlarge the thickness of CSR, which is against to pursue broadband absorber in deep-subwavelength dimension. The total thickness *L* in these cases are at [71.8, 81.2, 90.6, 100, 109.6, 119, 128.5, 138] mm corresponding to $w_1$ ranging from 7 to 14 mm with a step size of 1 mm, as illustrated by blue dashed line with symbols in Fig. 2d. As a compromise, to construct broadband absorber in sub-wavelength dimension, we can opt resonators array showing matched surface impedance at multiple frequencies. Figure 3a shows the absorptance for a system composed of the aforementioned CSR (with $w_1 =10 \, \hbox {mm}$, named as “Type-I” CSR). It is seen that the system achieves PA at 236 Hz in theory (solid line) and shows 99.2% absorptance at 239 Hz in experiment (circles), which confirms the matched surface impedance shown in Fig. 3b. In this work, the standard test method ASTM E1050-12 is adopted to conduct the measurements. To achieve working frequencies with wide enough variation, another two types of CSRs have been devised. Type-II CSR [see inset in Fig. 3c] with geometric parameters $L =100 \, \hbox {mm}$, $W =68 \, \hbox {mm}$ and $t_{\mathrm{x}}=t_{\mathrm{y}}=1.4 \, \hbox {mm}$ aims to extend the working frequency to lower range, while type-III CSR [see inset in Fig. 3e] with $L =100 \, \hbox {mm}$, $W =32 \, \hbox {mm}$ and $t_{\mathrm{x}}=t_{\mathrm{y}}=1.4 \, \hbox {mm}$ to higher range. This is because the increasing of *W* decreases the resonant frequency by elongating the propagation length of sound in the zigzag channel, and vice versa. Similarly, we have devised metasurfaces by type-II CSR and type-III CSR which show PA at 183 Hz ($L=\lambda /18.8$) when $w_{1} =11.4 \, \hbox {mm}$ and at 333 Hz ($L=\lambda /10.3$) when $w_{1} =8.6 \, \hbox {mm}$, respectively, as seen from Fig. 3c and e. In experiments, the absorbers based on type-II and type-III CSRs show 99.6% absorptance at 184 Hz and 97.4% at 338 Hz, respectively. Figure 3d and f depict corresponding surface impedances which are perfectly matched with that of air at resonances.

### Tunable resonant frequency

**Figure 4:**
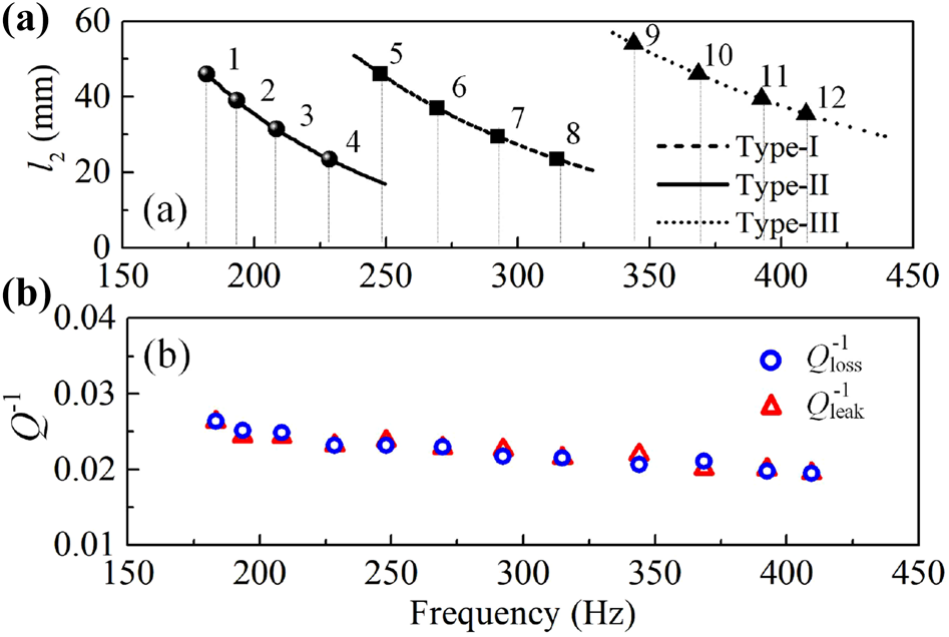
(**a**) Resonant frequency variation along with $l_2$. (**b**) Corresponding loss and leakage factors of the selected CSRs.

$Q_{\mathrm{loss}}^{-1}$ of CSR is dominantly derived from the channel 1 and $Q_{\mathrm{leak}}^{-1}$ is determined by the ratio of the slit area to the unit cell area ($Hw_1/S_{\mathrm{U}}$)^22^. Thus, to achieve matched impedance ($Q_{\mathrm{loss}}^{-1} =Q_{\mathrm{leak}}^{-1}$) at varied frequencies, the depth of the cavity ($l_2$) is tuned which hardly influences $Q_{\mathrm{leak}}^{-1}$ and $Q_{\mathrm{loss}}^{-1}$ of the system. We have investigated the resonant frequency of three types of CSRs on $l_2$ and opted 12 CSRs for constructing broadband absorber, as shown in Fig. 4a. The corresponding geometric parameters are listed in Table 1. Figure 4b presents the corresponding $Q_{\mathrm{loss}}^{-1}$ and $Q_{\mathrm{leak}}^{-1}$, which are highly matched and indicate that the matched impedances are achieved at this cases. Thereafter, a wide frequency range can be overlapped which provides the possibility to realize matched surface impedance at multiple frequencies in an integrated system. Hence, the individual CSRs for constructing broadband absorber have been determined.

**Table 1:** Geometric parameters of CSRs. (unit: mm).

	*L*	*W*	$w_1$	$t_x$	$t_y$	$l_2$		*L*	*W*	$w_1$	$t_x$	$t_y$	$l_2$
CSR_1	100	68	11.4	1.4	1.4	46	CSR_7	100	50	10	1.4	2.1	29.5
CSR_2	100	68	11.4	1.4	1.4	39	CSR_8	100	50	10	1.4	2.1	23.5
CSR_3	100	68	11.4	1.4	1.4	31.5	CSR_9	100	32	8.6	1.4	1.4	54
CSR_4	100	68	11.4	1.4	1.4	23.5	CSR_10	100	32	8.6	1.4	1.4	46
CSR_5	100	50	10	1.4	2.1	46	CSR_11	100	32	8.6	1.4	1.4	39.5
CSR_6	100	50	10	1.4	2.1	37	CSR_12	100	32	8.6	1.4	1.4	35.5

### Broadband absorber

**Figure 5:**
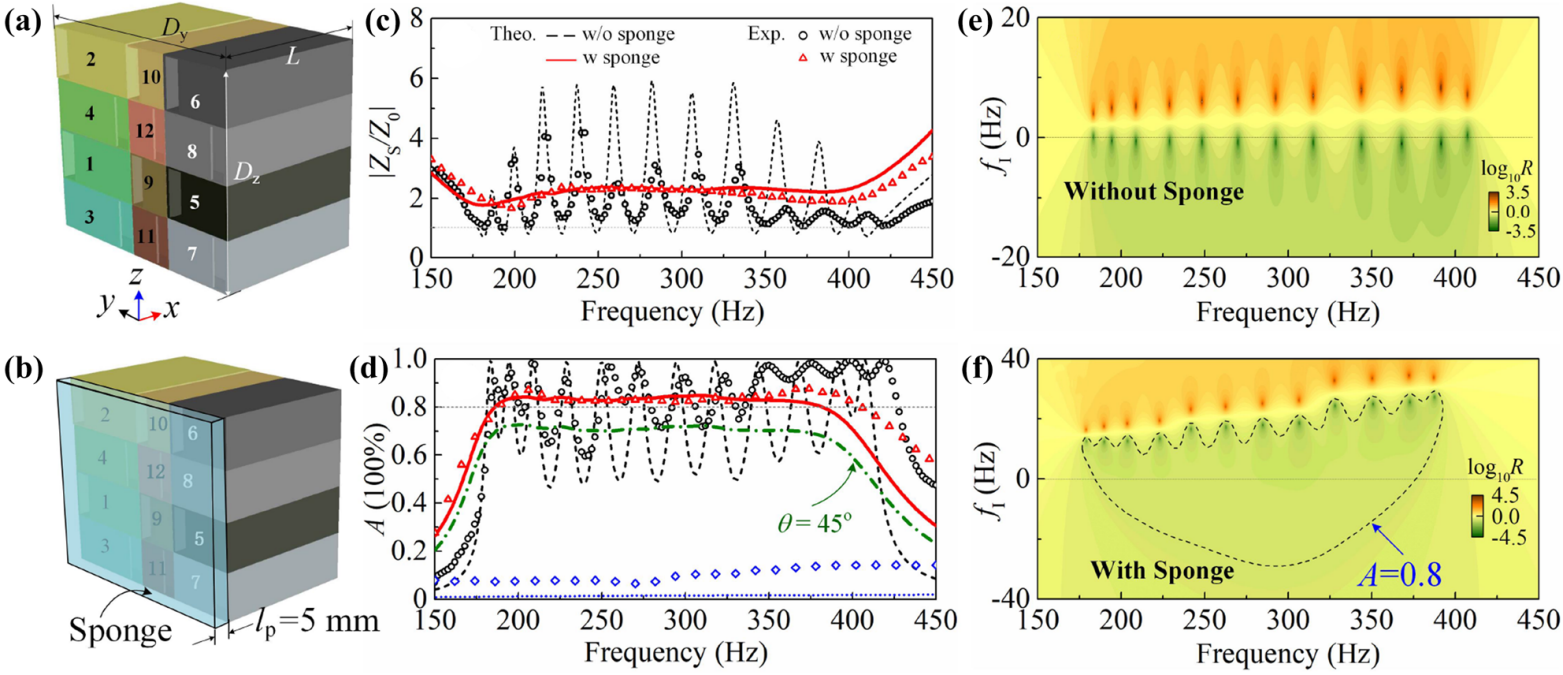
Schematics of (**a**) the multiband perfect absorber and (**b**) broadband absorber. (**c**) Absolute normalized surface impedance of the multiband absorber (black dashed line/circles) and broadband absorber (red solid line/triangles). (**d**) Absorptance of the multiband perfect absorber (black dashed line/circles) and broadband absorber (red dashed line/triangles). For comparison, the blue dotted line (squares) shows the theoretical (experimental) absorptance of only sponge layer backed by a rigid wall. (**e**) The ${\mathrm{log}}_{10}R$ distribution of system without sponge in complex frequency plane, the zeros of reflectance in real frequency axis confirm that the system is critically coupled. (**f**) The ${\mathrm{log}}_{10}R$ distribution of system with sponge in complex frequency. The dashed line indicates the absorptance of 0.8.

By employing the designed CSRs, a multi-frequency impedance-matching metasurface is constructed first, as schemed in Fig. 5a, where the middle, left and right columns are correspondingly type-I, type-II and type-III CSRs. Therefore, the periods in *y* and *z* directions are $D_{\mathrm{y}} =0.15 \, \hbox {m}$ and $D_{\mathrm{z}} =0.12 \, \hbox {m}$; the Arabic numbers from 1 to 12 denote for the resonators with different resonant frequencies from lowest to highest. To avoid the couplings due to the influence of spatial locations to the most extent, i.e., with absorptive peaks distributing evenly in frequency and demonstrating near-unity amplitudes, the CSRs with neighboring resonant frequencies are intentionally placed at intervals and the opening of CSRs are deliberately staggered. Designing this standalone metasurface aims to obtain moderate surface impedance and consequently, strengthens the coupling with sponge coating as illustrated in Fig. 5b. Figure 5c and d present the absolute impedances and absorptances (black dashed line and circles) of the system without sponge coating. Due to multi-mode resonances, the system shows matched impedances and achieves PA at the discrete resonant frequencies. Although relatively large surface impedances are formed by the anti-resonances between adjacent resonances, they are still significantly smaller than that of a rigid wall and present moderate impedance in a wide frequency range. Moreover, the thickness of absorber is still in sub-wavelength scale even at the 12th peak ($L=\lambda /8.4$ at 407.5 Hz).

Based on the moderate surface impedance of the multiband perfect absorber, the coupling with sponge coating can be enhanced. For the system shown in Fig. 5b, the surface impedance can be achieved by admittance-sum method^27^

3 $$\begin{aligned} {Y_{\mathrm{S}}=\sum _{i=1}^{12}{r}_{\mathrm{i}}{Y}_{\mathrm{i}}}, \end{aligned}$$

where $r_{\mathrm{i}}$ is the surface ratio. $Y_{\mathrm{i}}$ is the admittance of *i*-th CSR coated with sponge which can be expressed as

4 $$\begin{aligned} Y_{\mathrm{i}}=\frac{Y_{{\mathrm{r,i}}}+\mathrm{j}Y_{\mathrm{p}}\mathrm{tan}(k_{\mathrm{p}}l_{\mathrm{p}})}{1+\mathrm{j}Y_{{\mathrm{r,i}}}/Y_{\mathrm{p}}\mathrm{tan}(k_{\mathrm{p}}l_{\mathrm{p}})}, \end{aligned}$$

where $Y_{{\mathrm{r,i}}}$ is for the surface admittance of i-th CSR without sponge coating derived from transfer-matrix method and $Y_{\mathrm{p}}$ for the admittance of sponge coating (see “Methods” for details). Hence, the surface impedance is $Z_{\mathrm{S}}=1/Y_{\mathrm{S}}$ and absorptance follows

5 $$\begin{aligned} A = {\left| \frac{1-Y_{\mathrm{S}}z_0}{1+Y_{\mathrm{S}}z_0} \right| }^2, \end{aligned}$$

where $$z_0=\rho _0c_0/D_{\mathrm{y}}D_{\mathrm{z}}$$ being the specific impedance; $$\rho _0=1.21$$$$\mathrm{kg}/{\mathrm{m}}^3$$ and $$c_0 =343 \, \hbox {m/s}$$ are for the mass density and sound velocity of air. It is not unique that the thickness of sponge should be carefully decorated to supply proper viscosity in that Eq. (4) becomes $$Y_{\mathrm{i}}=Y_{{\mathrm{r,i}}}$$ with negligible thickness while turns into $$Y_{\mathrm{i}}=Y_{{\mathrm{p}}}$$ with excessive thickness.

With the coupling of sponge coating and backing metasurface, the system demonstrates much flatter surface impedance as shown in Fig. 5c, where the red solid line and triangles are for the theoretical and experimental results, respectively. It is found that the surface impedance at resonances increase with $l_{\mathrm{p}}$ as introduction of viscosity makes the system over-damped. However, the surface impedance at anti-resonances are more complicated since which are firstly decreased with $l_{\mathrm{p}}$ responsible for dissipating evanescent waves; when the evanescent wave components are efficiently dissipated, i.e.,$l_{\mathrm{p}} =5 \, \hbox {mm}$, the system can be characterized by a broadband hard boundary with $|{Z}_{\mathrm{b}}|=|{Z}_{\mathrm{S}}|\approx 2{Z}_{\mathrm{0}}$ and thus, continue to increase $l_{\mathrm{p}}$ will rise the surface impedance at anti-resonances instead. Hence, $l_{\mathrm{p}} =5 \, \hbox {mm}$ is selected in this work. The system shows absorptance over 80% in frequency ranges from $\sim$185 Hz to $\sim$385 Hz ($17.7 L_{\mathrm{t}}$ to $8.5 L_{\mathrm{t}}$ in wavelength) exceeding one octave (see Fig. 5d) and the relative bandwidth approaches to 70.2%. Compared with the absorptance of standalone backing metasurface without sponge coating, the low-efficiency absorptive valleys are ’erased’ and the average absorptance in frequency ranges from $\sim$185 Hz to $\sim$385 Hz is increased from 0.72 to 0.83, which can be ascribed to the existence of sponge coating can dissipate the evanescent sound components originated from anti-resonances^20,22,28,29^. Simultaneously, the sponge coating makes the original perfect absorptive peaks over-damped as displayed by the ${\mathrm{log}}_{10}R$ distribution in complex frequency plane (by introducing an imaginary frequency $f_{\mathrm{I}}$ into propagation constant), as shown in Fig. 5e and f. Without sponge coating, the zeros of reflectance lie in real frequency axis which confirms that the system is critically coupled at the specific resonant frequencies^30,31^. However, the zeros of reflectance moves above the real frequency axis in case with sponge coating, indicating the system being over-damped; namely, the sponge coating introduces extraordinary loss and breaks the original critical coupling conditions. Note that the absorptance $A=0.8$ along the dashed line in Fig. 5f, and $A>0.8$ in the region inside the contour.We emphasize that the resonant frequencies of CSRs should be carefully decorated since widen resonant frequency interval will decrease the absorptance at anti-resonances.

Moreover, we have investigated the absorptive performance for obliquely incident sound, which demonstrates near 0.7 absorptance even at $\theta =45 ^{\circ }$ as illustrated by the olive dash dotted line in Fig 5d. Here, $\theta$ stands for the intersection angle between the incident sound wave and the normal direction of the absorber, i.e., $\theta =0 ^{\circ }$ denotes for normal incidence. For reference, the absorptance of the system composed of a sponge coating backed by a rigid wall is presented (blue dotted line and symbols) in Fig. 5d. It can be seen that such system has little absorption. To sum up, by substituting the well-designed CSRs arrays into rigid wall, the absorptive performances of the ultrathin sponge can be largely enhanced to achieve ultra-wide absorptance in deep-subwavelength scales.

**Figure 6:**
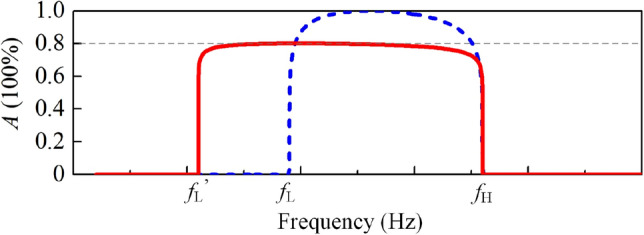
The broadband absorptance at unity (blue dashed line) and at 0.8 (red solid line) in causality principle.

## Discussion

In conclusion, we have exposed the absorptive mechanism of sound porous materials with a backing plate, which proves that higher-efficiency absorptions can be achieved with more moderate-impedance boundary. Hence, backed by a well-designed metasurface, an ultrathin sponge can demonstrate excellent absorptance. We emphasize that the mechanism presents universality and demonstrates superiority in many other fields, i.e., designing broadband absorbers for waterborne sound since acoustically softer materials (than water) can be designed more easily or even served by non-resonance materials. Then, the connections between the CMT and effective boundary theory has been built up, which indicates that the under-damped (over-damped) system is characterized to be a soft (hard) boundary. Additionally, we also illustrate that getting softer sound boundary requires thicker structure, which is against to construct deep-subwavelength sound absorber. Therefore, we exploit a different route to build the broadband absorber by coupling the sponge with a multiband impedance-matched metasurface, instead of a softer one in Ref. 22. The constructed broadband sound absorber demonstrates >80% absorptance over one octave. To achieve wide range of working frequencies, we have devised three types of CSRs with different width of channels. More importantly, the thickness of absorber is still deep-subwavelength when compared to the wavelength at higher cut-off frequency. Differing from the absorber which couples a sponge coating with a soft (under-damped) boundary in ref.^22^, the backed plate in this work is critically coupled so that broader band (228 Hz to 319 Hz and relative bandwidth at 33.2% in ref.^22^) can be achieved with thinner structure (total thickness is 125 mm in ref.^22^). Regarding causality theory^20^, if $Z/Z_0=a$ when $\omega >\omega _{\mathrm{L}}=2\pi f_{\mathrm{L}}$ and $\omega <\omega _{\mathrm{H}}=2\pi f_{\mathrm{H}}$ while $Z/Z_0=0$ in remaining frequencies, the surface impedance can be expressed as $Z=(a\pi Z_0)/(\pi -2a\mathrm{j}\mathrm{tanh}(\omega _{\mathrm{L}}/\omega )+2a\mathrm{j}\mathrm{tanh}(\omega /\omega _{\mathrm{H}}))$. In case of $a=1$ (the case in ref.^22^), the absorptance from $f_{\mathrm{L}}$ to $f_{\mathrm{H}}$ are at unity as illustrated by the blue dashed line in Fig. 6. However, if we set the absorptance at 0.8 ( $a=(5+\sqrt{5})/(5-\sqrt{5})$ with the same upper cut-off frequency, the lower cut-off frequency ($f_{\mathrm{L}}^{'}<f_{\mathrm{L}}$) decreases as illustrated by the red solid line. Hence, the bandwidth of absorptance can be broadened. The broadband, high-efficiency, and subwavelength characteristics of the absorber may prompt versatile applications in environmental acoustics and room acoustics.

## Methods

### Theoretical model of sponge layer

The sponge layer is characterized by the Johnson-Champoux-Allard model for porous materials, where the effective mass density^32^ and compressibility modulus^33^ can be expressed as

6 $$\begin{aligned} \rho _{p}&=\rho _0\alpha (\omega )/\phi , \end{aligned}$$

7 $$\begin{aligned} C_{p}&=\frac{\gamma P_{0}}{\gamma -{(\gamma -1)/\alpha _{\tau }(\omega )}}. \end{aligned}$$

Here, $\gamma$ is the specific heat ratio. $\alpha (\omega )$ is the dynamic tortuosity and $\alpha _{\tau }(\omega )$ is the thermal tortuosity given by

8 $$\begin{aligned} \alpha (\omega )&=\alpha _{\infty }\left( 1+j\frac{\omega _{\nu }}{\omega }\sqrt{1-j\eta \rho _{0}\omega \left( \frac{2\alpha _{\infty }}{\sigma \phi \varLambda }\right) ^2} \right) , \end{aligned}$$

9 $$\begin{aligned} \alpha _{\tau }(\omega )&=1+j\frac{\omega _{v}^{'}}{\mathrm{Pr}\omega }\sqrt{1-j\eta \rho _0\omega {\left( \frac{2\alpha _{\infty }}{\sigma ^{'}\phi \varLambda ^{'}}\right) }^2}, \end{aligned}$$

where $\omega _{v}=\sigma \phi /\rho _0\alpha _{\infty }$ is angular Biot frequency; $\omega _{c}^{'}=\sigma ^{'}\phi /\rho _0\alpha _{\infty }$ is the adiabatic cross-over angular frequency; Pr is the Prandtl number and $\eta$ is the dynamic viscosity of the fluid. The other parameters describe the properties of the porous material, including the tortuosity $\alpha _{\infty }$, porosity $\phi$, flow resistivity $\sigma$, viscous characteristic length $\varLambda$, thermal characteristic length $\varLambda ^{'}$ and thermal resistivity $\sigma ^{'}(=8\alpha _{\infty }\eta /\phi \varLambda ^{'})$. The porous material used in this work is Melamine foam with acoustical parameters listed in Table 2. Therefore, the effective propagation constant and impedance can be given as $k_{\mathrm{p}}=\omega \sqrt{(\rho _{\mathrm{p}} C_{\mathrm{p}})}$ and $Z_{\mathrm{p}}=\sqrt{(\rho _{\mathrm{p}}/C_{\mathrm{p}} )}$, respectively.

**Table 2:** Acoustical parameters of the porous materials.

	$\phi$	$\alpha _{\infty }$	$\varLambda ({\mu } \, \mathrm{m})$	$\varLambda ^{'}\, ({\mu } \, \mathrm{m})$	$\sigma ({\mathrm{N s m}}^{-4})$
Melamine foam	0.95	1.42	180	360	7800
Polyurethane foam	0.96	1.07	273	672	2843
Wool	0.94	1.03	56	85	28500

**Figure 7:**
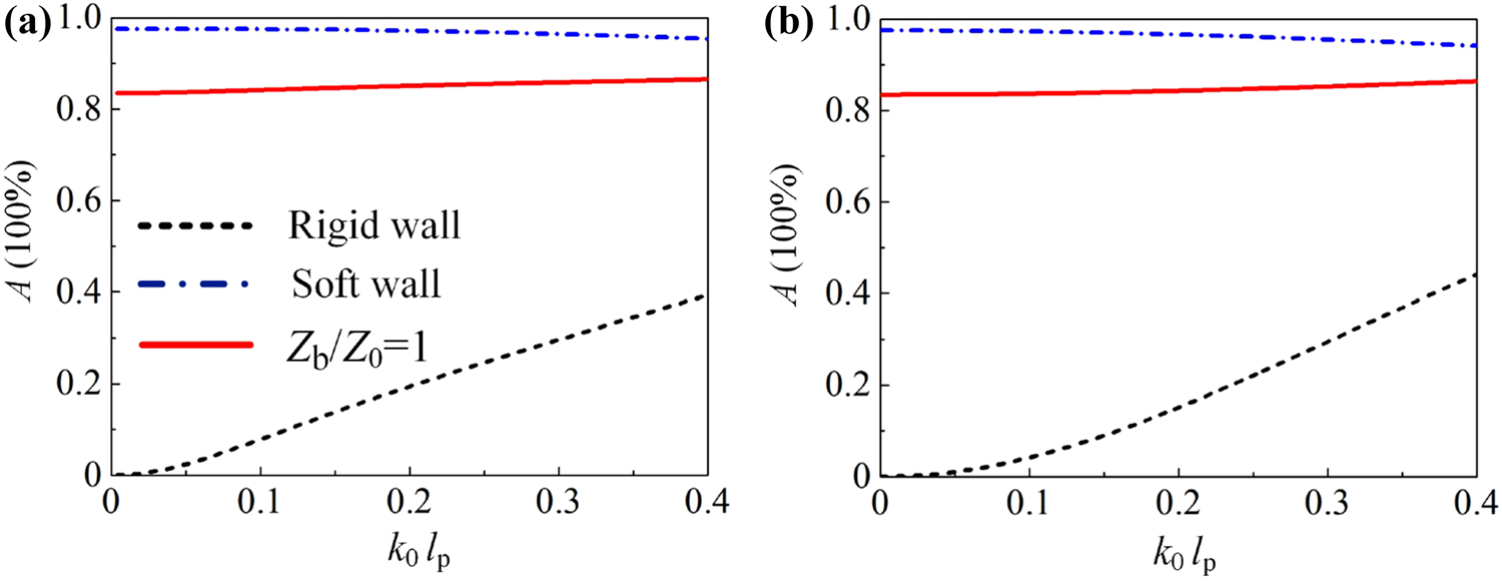
(**a**) and (**b**) are absorptance for polyurethane foam with low flow resistivity and wool with high flow resistivity, respectively.

Although the result in Fig. 1d is derived from melamine foam with specific thickness at 0.05 m, the characteristics can be generalized. Here, polyurethane foam with lower flow resistivity and wool with higher flow resistivity are employed^34^; the corresponding acoustical parameters are listed in Table 2. Figure 7a and b present the absorptance with melamine foam ( $l_{\mathrm{p}}=0.2 \, \hbox {m}$) and wool ($l_{\mathrm{p}}=0.02 \, \hbox {m}$), respectively. Generally, to achieve the similar result as shown in Fig. 1d, the required thickness will be smaller (larger) with higher (lower) flow resistivity porous materials to supply roughly same viscosity.

### Theoretical model of CSR

According to the Stinson model^35^, the acoustical character of a tube with rectangular cross section can be described by complex density $\rho _{\mathrm{e}}^{\mathrm{k}}$ and compressibility $C_{\mathrm{e}}^{\mathrm{k}}$ (k stands for *k*-th channel of CSR) given as

10 $$\begin{aligned} \rho _{\mathrm{e}}^{\mathrm{k}}&=\rho _0{\frac{v\omega _{\mathrm{k}}^2H^{2}}{64\mathrm{j} \omega }}\left\{ {\sum _{m=0}^{\infty }\sum _{n=0}^{\infty } \left[ {\alpha _{\mathrm{m}}^2\beta _{\mathrm{n}}^2 \left( \alpha _{\mathrm{m}}^2+\beta _{\mathrm{n}}^2+\frac{\mathrm{j}\omega }{v}\right) }^{-1}\right] }\right\} ^{-1}, \end{aligned}$$

11 $$\begin{aligned} C_{\mathrm{e}}^{\mathrm{k}}&={\frac{1}{P_0}}\left\{ {1-{\frac{64\mathrm{j}(\gamma -1)}{v^{'}w_{\mathrm{k}}^2H^2}{\sum _{m=0}^{\infty }\sum _{n=0}^{\infty }{\left[ {\alpha _{\mathrm{m}}^{2}\beta _{\mathrm{n}}^{2} \left( \alpha _{\mathrm{m}}^{2}+\beta _{\mathrm{n}}^{2}+\frac{\mathrm{j}\omega \gamma }{v^{'}}\right) } \right] }^{-1}}}}\right\} , \end{aligned}$$

where the constants $\alpha _{\mathrm{m}}=(2m+1)\pi /w_{\mathrm{k}}$ and $\beta _{\mathrm{n}}=(2n+1)\pi /H$; $v=\mu /\rho _{0}$ and $v^{'}=\kappa /(\rho _{0} C_{\mathrm{v}})$ with $\mu =1.814\times 10^{-5} \, \hbox {Pa s}$, $\kappa =2.624\times 10^{-2} \, \hbox {W}/(\mathrm{m}\times \mathrm{K})$ and $C_{\mathrm{v}}=0.7178\times 10^{3} \, \mathrm{kJ}/((\mathrm{kg}\times \mathrm{K}))$ denoting the kinematic viscosity, thermal conductivity and specific heat of air, respectively; $P_{0}=1.013\times 10^{5} \, \hbox {Pa}$ and $\gamma =1.4$ are atmosphere pressure and the ratio of specific heat, respectively. $\mathrm{j}=\sqrt{-1}$ is the imaginary unit. Hence, the effective propagation constant and specific impedance in the channels can be deduced as $k_{\mathrm{e}}^{\mathrm{k}}=\omega \sqrt{\rho _{\mathrm{e}}^{\mathrm{k}}C_{\mathrm{e}}^{\mathrm{k}}}$ and $z_{\mathrm{e}}^{\mathrm{k}}=\sqrt{\rho _{\mathrm{e}}^{\mathrm{k}}/C_{\mathrm{e}}^{\mathrm{k}}}/S_{\mathrm{k}}$ .

The transfer matrix which relates the sound pressures and velocities at the entrance and terminal of CSR is defined as $\mathbf{M }_{\mathrm{CSR}}=\mathbf{M }_{\mathrm{channel,1}} \mathbf{M }_{\mathrm{channel,2}}$. Here $$\mathbf{M }_{\mathrm{channel,k}}=\begin{bmatrix} \mathrm{cos}(k_{\mathrm{e}}^{\mathrm{k}}l_{\mathrm{k}})&{}\mathrm{j}z_{\mathrm{e}}^{\mathrm{k}}\mathrm{sin}(k_{\mathrm{e}}^{\mathrm{k}}l_{\mathrm{k}})\\ \mathrm{j}\mathrm{sin}(k_{\mathrm{e}}^{\mathrm{k}}l_{\mathrm{k}})/z_{\mathrm{e}}^{\mathrm{k}}&{}\mathrm{cos}(k_{\mathrm{e}}^{\mathrm{k}}l_{\mathrm{k}}) \end{bmatrix}$$ is the transfer matrix of the *k*-th channel. Moreover, the end correction length $\varDelta l_{\mathrm{slit}}=w_{1} \phi _{\mathrm{t}}\sum _{n=1}^{\infty }{\frac{\mathrm{sin}^{2}(n\pi \phi _{\mathrm{t}})}{({n\pi \phi _{\mathrm{t}}})^{3}}}$ is introduced to describe sound pressure radiation at the discontinuity from the channel to free space, where $\phi _{\mathrm{t}}=w_{1}/W$ is the ratio of slit to the width of the structure. Hence, the transfer matrix of the slit can be expressed as $$\mathbf{M }_{\mathrm{slit}}=\begin{bmatrix} {1}&{}jz_{\mathrm{e}}^{1}k_{\mathrm{e}}^{1}\varDelta l_{\mathrm{slit}} \\ {0}&{}{1} \end{bmatrix}$$. Consequently, the total matrix can be deduced as

12 $$\begin{aligned} \mathbf{M }_{\mathrm{total}}=\mathbf{M }_{\mathrm{slit}}\mathbf{M }_{\mathrm{CSR}}=\mathbf{M }_{\mathrm{slit}} \mathbf{M }_{\mathrm{channel,1}} \mathbf{M }_{\mathrm{channel,2}}. \end{aligned}$$

Due to the rigid boundary, the particle velocity is zero at terminal, i.e., $v_{\mathrm{terminal}}=0$, and hence, the specific admittance at the entrance of the CSR can be derived as

13 $$\begin{aligned} Y_{\mathrm{CSR}}=\mathbf{M }_{\mathrm{total}}(2,1)/\mathbf{M }_{\mathrm{total}}(1,1). \end{aligned}$$

By substituting the expression into Eqs. (3-5), the absorptance can be obtained.
